# Knowledge, attitude and practice of physicians and nurses at the cape coast teaching hospital in the Central Region of Ghana on spontaneous adverse drug reaction reporting

**DOI:** 10.1371/journal.pone.0288100

**Published:** 2023-07-07

**Authors:** Julianne Frimpomaa Powell, Isaac Tabiri Henneh, Martins Ekor

**Affiliations:** 1 School of Medical Sciences, College of Health and Allied Sciences, University of Cape Coast, Cape Coast, Ghana; 2 Department of Pharmacotherapeutics and Pharmacy Practice, School of Pharmacy and Pharmaceutical Sciences, College of Health and Allied Sciences, University of Cape Coast, Cape Coast, Ghana; 3 Department of Pharmacology, School of Medical Sciences, College of Health and Allied Sciences, University of Cape Coast, Cape Coast, Ghana; Cukurova University: Cukurova Universitesi, TURKEY

## Abstract

Ghana’s rate of reporting adverse drug reaction (ADRs) over the past years has consistently been below the WHO standard despite utilizing the spontaneous or voluntary reporting system. While underreporting undermines the pharmacovigilance system and poses a huge threat to public health safety, there is limited information on the perspectives of healthcare workers directly involved in drug administration. The present study investigated the knowledge, attitude and practice of physicians and nurses at the Cape Coast Teaching Hospital (CCTH) towards spontaneous reporting of ADRs (SR-ADRs). A descriptive cross-sectional survey was employed in the study. Pre-tested (Cronbach’s alpha value of 0.72) and validated questionnaires comprising 37 open-ended and close-ended questions were administered to 44 doctors and 116 nurses at the CCTH who had been practicing for at least six months prior to study. Out of the 160 administered questionnaires, 86 was administered face-to-face and the remaining via e-mails. Descriptive analysis was performed and the results were presented in simple frequencies and percentages. Binary logistic regression model was used to test association of the independent variables with SR-ADRs. With a response rate of 86.4% for physicians and 59.5% for nurses, 38 (35.5%) physicians and 69 (64.5%) nurses completed the questionnaires and returned same. Majority (82.3%, 88) of the respondents knew that it is their responsibility to report ADRs although their knowledge levels was found to be inadequate (that is ≤80%) in majority (66.7%) of the text items that assessed knowledge levels. On the attitude of respondents, it was found that 57% (61) of them agreed that under-reporting was due to complacency whereas 80.4% (86) of them agreed that it was due the lack of adequate training. On the issues of practice, the prevalence of encountering, assisting in the management, and reporting of ADRs were 26.1% (28), 17.8% (19) and 7.5% (8) respectively. Also, nurses were 1.22 times more likely to encounter a patient with ADRs and twice more likely to fill and forward ADR form than doctors during management. Respondents with more than six months but less than one year of practice experience were more likely (AOR = 1.38, 95% CI: 2.72–7.3) to encounter a patient with ADRs as compared to those with just six months of practice experience. Furthermore, male respondents were more likely (AOR = 2.42, 95% CI: 1–5.85) to encounter patients with ADRs but less likely (AOR = 0.49, 95% CI: 0.91–2.6) to fill and forward ADR form compared to their female counterparts. In conclusion, doctors and nurses at the CCTH had inadequate knowledge about ADRs and its existing pharmacovigilance systems, thus accounting for the low spontaneous ADRs reporting in the facility.

## Introduction

The World Health Organization (WHO) defines adverse drug reaction (ADR) as an undesirable and unexpected reaction to an over-the-counter, prescription or pharmaceutical medication at a dosage normally used to prevent, diagnose, treat or modify disease or physiological function [1, 2]. Adverse drug reactions (ADRs) are becoming more prevalent in recent times and increasingly advancing into a global health issue that requires the attention of all stakeholders, regardless of practice settings [3]. ADRs have been identified as a leading cause of morbidity and mortality in people of all ages, resulting in a huge number of hospitalizations and a significant financial burden on society and healthcare systems globally [4, 5]. Recent reports show that ADRs have significantly increased drugs, laboratory and hospitalization cost of patients [5]. A cross-sectional study on mortality from ADRs in adult hospitalized patients conducted in four South African hospitals revealed that ADRs are associated with 2.5%– 18% of deaths of patients [6]. It was also found from the same study that 43% of ADRs were considered preventable, hence recommendations have consistently been made to strengthen systems for health care worker training and support. It is appropriate to infer that correcting underreporting of ADRs in Ghana, as well as globally would go a long way to eliminating several preventable deaths, particularly those resulting from adverse drug reactions.

In spite of its damning implications on public health and safety, available evidence suggests that professionals in the healthcare sector, particularly in developing countries, do not report many adverse reactions, due to a number of factors which include a lack of awareness and understanding of pharmacovigilance and adverse reaction reporting systems [7, 8]. The World Health Organization (WHO) has defined pharmacovigilance as “the science and activities relating to the detection, assessment, understanding and prevention of adverse effects or any other medicine/vaccine related problem” [9, 10]. Pharmacovigilance activities in Ghana are coordinated by the Ghana’s Food and Drugs Authority (FDA). As the 65^th^ member of the WHO International Drug Monitoring Programme and the first country in West Africa, Ghana joined in November 2001. Reports of adverse reactions to the FDA are mainly generated by healthcare professionals and marketing authorization holders (MAHs). The FDA serves as a repository for reports. To coordinate its activities, nine Regional Offices with dedicated Regional Pharmacovigilance Officers were assigned to facilitate reporting of ADRs under the FDA’s decentralized Pharmacovigilance (PV) system [11]. In Ghana, reporting ADRs is the responsibility of health professionals. This responsibility is backed by the Public Health Act 2012, Act 851, Part 7, Section 125. To report a suspected ADR for products marketed in Ghana, Health care Professionals should complete a copy of the Adverse Reaction Reporting Form which could be obtained from all Regional Offices of Authority or the Safety Monitoring Department of the Food and Drugs Author or healthcare institutions. The completed report may be delivered to the Food and Drugs Authority Head Office in Accra or through the Regional Offices [11].

Spontaneous ADR reporting (SADRR) refers to a process of collecting information on suspected cases of adverse drug reaction from individual patients, with the primary objective of detecting unknown potential toxicity from drugs [12–14]. The formulation of this method dates back to the 1960s in response to a delay of five years in the recognition of the link between thalidomide exposure during pregnancy and a rare congenital limb deformity known as phocomelia [15]. SADRR has become a global phenomenon and a cornerstone of pharmacovigilance [16]. In this regard, all professionals in the healthcare sector are crucial to the pharmacovigilance system, and as such are required to possess considerable knowledge and expertise in medication safety, as well as being able to recognize, detect, manage and report ADRs early [17].

Recent studies still provide evidence of underreporting of ADRs in Ghana despite several efforts to increase spontaneous ADR reporting in the country. The underreporting has not only disrupted efforts aimed at detecting, assessing, and preventing several rare, severe, and unknown ADRs, but also undermined their burden in the population. Subsequently, this has led to an increase in medication-induced illnesses and disabilities [18, 19]. To the best of our knowledge, studies assessing the knowledge and attitudes of Ghanaian health workers to spontaneous ADR reporting and how these influence their practice are very scanty. The dearth of information in this regard makes it difficult to know how the country is faring in the aspects of detection, management, documentation, and reporting of ADRs, which are essential in ensuring patient safety. Although we are aware of few studies conducted in some Ghanaian hospitals in urban areas like Greater Accra [18, 19] and Volta Region [20], there is still a huge dearth of information on ADR reporting in Ghana. Importantly, to the best of our knowledge, no study has been conducted in the Central Region of Ghana, which is the setting for the current study. In this study therefore, we determined the knowledge, attitude, and practice of nurses and physicians (the two main categories of healthcare workers involved in the administration of medications) at the Cape Coast Teaching Hospital of Ghana in relation to spontaneous reporting of ADRs.

## Methodology

### Study area

The research was conducted at the Cape Coast Teaching Hospital (CCTH), a 400-bed capacity referral hospital in the Central Region of Ghana. The population of Cape Coast is estimated to be 189,925 according to the 2021 Population and Housing Census [21]. The hospital offers a variety of outpatient and inpatient services including family medicine, general medicine, accident and emergency, and wound care. A wide range of specialist services are offered, including surgery, internal medicine, pediatrics and child health, obstetrics and gynecology, oncology, radiology, dentistry, orthopedics etc.

### Study design and population

The study employed a descriptive cross-sectional survey design. Pre-tested and validated questionnaires comprising open-ended and close-ended questions were formulated and administered to physicians and nurses at the Cape Coast Teaching Hospital who had been practicing for at least six months prior to the study. At the time of data collection, there were 266 doctors and 809 nurses at post in the facility.

### Inclusion and exclusion criteria

The healthcare professionals included in this study were the nurses and physicians who constitute a major workforce in the healthcare industry. Nurses were considered because they are major stakeholders in the healthcare team with the responsibility of administration of drugs ordered by a registered medical practitioner as well as issues relating to pharmacovigilance [21]. The nurses are responsible for administering oral and rectal medications; performing hypodermic, intramuscular, and intravenous injections; applying medicine to the eyes, ears, throat, vagina, urethra, and skin [22]. Similar to some aspects of pharmacists’ roles, nurses can prepare and dispense medications through crushing of pills and preparing measured quantities of drugs for injections [23–25]. In addition to nurses, physicians were also included because they constitute another important profession in the healthcare team with the responsibility of prescribing and in some cases administering medications to patients in healthcare settings and as such, have an important role to play in pharmacovigilance.

It was also ensured that participants should have practiced for a minimum of six months prior to the study. Again, consent to participate was an inclusion criterion.

Although pharmacists play an important role in pharmacovigilance as reported in an earlier study by Shrestha et al. [22], they were excluded from this present study because of their limited numbers in the study setting. Other members of the healthcare team who were not directly involved in the prescription, administration and dispensing of drugs were excluded.

#### Sample size and sampling procedure

Based on ADR reporting prevalence of 17.3% [18] and 20% [19] in Ghana, a prevalence of 20% was assumed for this study. Also, a z-value corresponding to 95% level of significance, and an absolute permissible error of 5% were assumed in the sample size estimation in this study using the Cochran’s formula for sample size determination [26]. A sample size of 142 healthcare workers consisting of 35 doctors and 107 nurses was therefore required. To cater for non-response of some participants, researchers administered 160 questionnaires to 44 doctors and 116 nurses.

### Data collection procedure

A convenience and purposive sampling methods were adopted. Both face-face-face and electronic data collection procedures were used.

#### Face-face-data collection

Eighty-six (86) hard copies of the questionnaires were administered to 24 physicians and 38 nurses in the various departments of CCTH The aim of the study, its benefits and possible disquiets were explained to the participants and an informed consent was obtained from each participant prior to administering the questionnaires. Evidence of this consent was a signature of the participant on the hard copy of the questionnaire. The participants were allowed about two weeks within which the questionnaires were collected by the researcher upon completion. The data collection was conducted within a month, from 1^st^ July, 2020 to 31^st^ July, 2020. Data were collected at the Out-Patient Department, Emergency Ward, and other wards of the CCTH. Each questionnaire took about twenty minutes to complete after which they were later collected directly by the Principal Investigator. In all, 59 of the respondents completed and submitted their responses. Out of this number, 21 were physicians and 38 were nurses.

#### Electronic data collection procedure

Due to the restrictions that accompanied COVID-19 pandemic within the study period, it was necessary to adopt an on-line administration of questionnaires. This was done in keeping with the Checklist for Reporting Results of Internet E-Surveys (CHERRIES) [27]. As such, softcopies of the questionnaires were designed using Google forms and subsequently administered to 20 physicians and 54 nurses through their e-mail addresses. Evidence of their consent was a signature of the checking of the “I agree box”. In all, 48 complete responses were obtained from 17 physicians and 31 nurses.

#### Merging of face-to-face and electronic data

To merge the hard copy data and the online data, one of the authors keyed in all the hard copy data into the Google form to generate a single Excel file. A second author crosschecked the Excel data for errors and the file was exported to IBM’s SPSS software for analysis.

### Data collection instrument

The study utilized closed-ended structured questionnaires (S1 Appendix) which comprised the following sections: the demographics of the participants, information on knowledge of reporting ADRs, attitudes towards reporting ADRs, factors that were perceived to contribute to under-reporting and the level of education and training on reporting of participants. Also, participants’ level of knowledge of ADR reporting, their understanding of ADR reporting and its purpose, awareness of who should report ADRs, awareness of the existence of pharmacovigilance centers and awareness of the existence of National ADR reporting forms, the proportion of health workers who had witnessed ADRs in the management of patients (in-patient and out-patient) in the past 6 months at the Cape Coast Teaching Hospital and the proportion who had reported this to a drug regulatory authority were also assessed. The questionnaires were self-designed using information from earlier studies and contextualized into local situations [3, 19]. In all, there were 37 items in the questionnaire administered.

### Validation of survey questionnaire

A validity test was performed before the survey was implemented to ensure that the questionnaire has face and content validity. In order to assess the questionnaire’s clarity, relevance, and ease of understanding, two professors from the School of Medical Sciences, University of Cape Coast were contacted for their inputs. We then finalized the questionnaire based on their feedback. Pilot tests were conducted on ten doctors and ten nurses before the actual survey to pre-test the questionnaire. In order to assess the reliability of the questionnaire, Cronbach’s coefficient alpha was calculated. It had a 0.72 internal consistency.

### Data processing and analysis

Data obtained from the questionnaires were analyzed using IBM SPSS version 23 software and Stata version 14. Study participants were profiled using descriptive statistics for their socio-demographic characteristics. Categorical variables were analyzed using simple frequencies and percentages. KAP score was computed to determine the knowledge levels of participants using an earlier described method [28]. An analysis of logistic regression was used to determine the association between the independent variables (adjusted odds ratio) and the dependent variable (spontaneous reporting of ADRs). Statistical estimates were expressed with 95% Confidence Intervals (CIs) and a *p* value less than 0.05 was considered statistically significant in the Chi-square test.

### Ethical consideration

Approval for the study was obtained from the University of Cape Coast Institutional Review Board (ethical clearance ID: UCCIRB/CHAS/2019/155) and the Cape Coast Teaching Hospital Ethical Review Board (ethical clearance-ref: CCTHERC/EC/2020/026) prior to the commencement of the study. Informed consent was obtained from participants before including them in the study. Persons who did not consent to enrolling as participants were excluded from the study. Participants were informed that their data would be managed in keeping with the standard requirement of ethical practice. They were also informed of their right to withdraw from the study at any point in the research. It was ensured that every statement in the final write up was appropriately referenced.

## Results

### Demographic data of respondents

As presented in Table 1, one hundred and sixty (160) questionnaires were administered to doctors (n = 44) and nurses (n = 116) in the hospital. With a response rate of 86.4% for doctors and 59.5% for nurses, 38 (35.5%) of the respondents who completed the questionnaires and returned same were doctors and 69 (64.5%) of them were nurses. Also, out of the 160 administered questionnaires, 86 was administered face-to-face and the remaining via e-mails. The response rates were 68% (59 out of 86) and 64% (48 out of 74) respectively for face-to-face and online methods of questionnaire administration. There were more female respondents (60.7%) than male respondents. Also, almost half of the respondents had practiced between one to five years (49.5%) and those who had practiced for more than five years were 21.5%.

**Table 1:** Demographic data of respondents.

Variable	Category	n (%)
Age	18–25	17 (15.9)
	26–35	72 (67.3)
	36–45	16 (15.0)
	46–55	2 (1.9)
Sex	Female	65 (60.7)
	Male	42 (39.3)
Occupation	Doctor	38 (35.5)
	Nurse	69 (64.5)
Period of clinical practice	6 months	12 (11.2)
	>6months but < 1 year	19 (17.8)
	1 year—5 years	53 (49.5)
	6 years-10 years	18 (16.8)
	>10 years	5 (4.7)

N = 107

### Knowledge on reporting ADRs

As presented in Table 2, it was found from the study that majority (82.3%) of the respondents either agreed or strongly agreed to the assertion that it is the responsibility of health workers to report ADRs. Similarly, majority (98%) of the respondents also either agreed or strongly agreed that patients could report on their ADRs. Furthermore, it was revealed that most (65.1%) of the respondents either agreed or strongly agreed to the suggestion that reporting of suspected and serious ADRs should be within seven (7) days. However, almost half of the respondents (43.9%) did not have knowledge about the existence of ADR reporting scheme in the hospital and where they could submit the already filled forms.

**Table 2:** Respondents’ knowledge levels on ADRs reporting.

**Knowledge on reporting ADRs**	**Agree n (%)**	**Disagree n (%)**	**Remark**
Health workers are to report ADRs (N = 107)	88 (82.3%)*	19 (17.7%)	Adequate
Patients can report ADRs (N = 106)	98 (91.6%)*	9 (8.4%)	Adequate
Reporting suspected and serious ADRs should be within 7 days (N = 106)	69 (65.1%)*	37 (34.9%)	Inadequate
I have knowledge of availability of ADR reporting (N = 106)	60 (56.6%)*	46 (43.4%)	Inadequate
I am Aware of where to access ADR forms in my institution (N = 106)	72 (67.9%)*	34 (32.1%)	Inadequate
I know of where to return the fully-filled ADR reporting (N = 106)	60 (56.6%)*	46 (43.4%)	Inadequate
**Overall cut-off for knowledge score**	**Frequency (%)**		**Remark**
Score > 80	2 (33.3%)		Adequate
Score ≤ 80	4 (66.7%)		Inadequate

*Appropriate response, Maximum obtainable score = 6, Percentage knowledge score = (individual score/6)×10

### Respondents’ attitudes towards spontaneous ADRs reporting

On the attitudes of respondents with regards to under-reporting of ADRs as presented in Table 3, it was found that 57% (61) of them agreed that it was due to complacency of health workers whereas 80.4% (86) of them agreed that it was due the lack of adequate training. However, high percentage of the respondents (66.4%) disagreed with the assertion that health workers avoid ADRs reporting in order to avoid legal issues. It was also found that most of the respondents (82.7%) disagreed that low reportage of ADRs was due to insignificant number of such issues.

**Table 3:** Respondents’ attitudes towards spontaneous ADRs reporting.

Attitude of physicians and nurses	Agree n (%)	Disagree n (%)
Under-reporting is due to complacency on part of health workers (N = 107)	61 (57%.0)	46 (43.0)
Under-reporting by health workers is to avoid legal issues (N = 107)	36 (33.6%)	71 (66.4)
Underreporting is due to insignificance of ADR issues (N = 104)	18 (17.3%)	86 (82.7)
Underreporting is due to lack of training on reporting technique (N = 104)	86 (80.4%)	21 (19.6)
Underreporting is due to lethargy on part of health workers (N = 104)	47 (45.2%)	57 (54.8)

### Respondents ADR practices in the past six months

**Fig 1:**
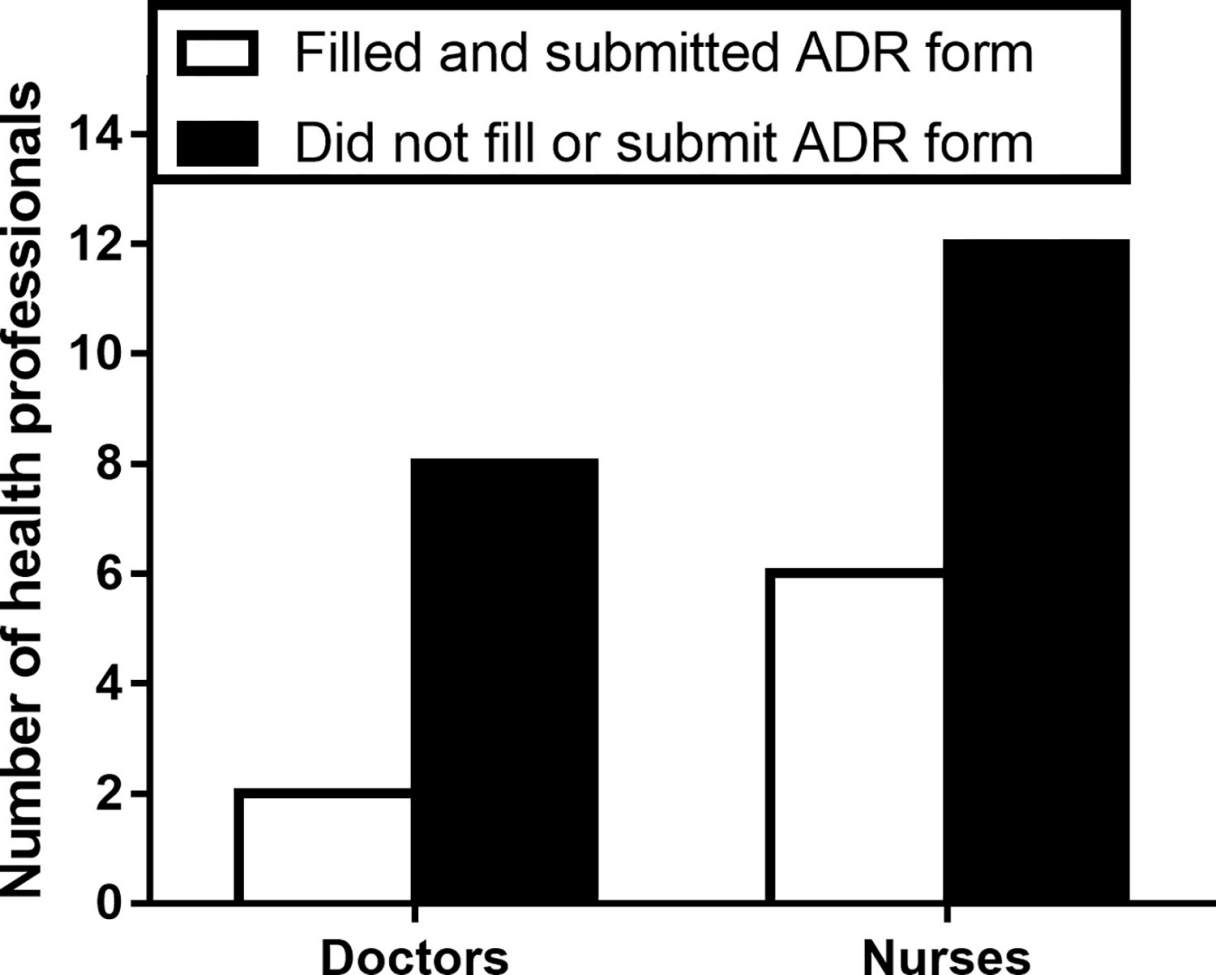
Distribution of health professionals who filled and forwarded the ADR forms during management of patients.

In Table 4 and Fig 1, respondents were asked to indicate their experiences with respect to adverse drug reaction reporting in their health facility in the past six months. It was found that 28 (26.2%) out 107 respondents indicated that they had encountered ADR at the CCTH prior to the study. Out of the 28 respondents who had encountered ADR in the facility within the past 6 months, 19 (67.9%) of them were involved in the management of the ADR whiles only 8 (28.6%) of them filled and forwarded the ADR form. This implies that the prevalence of encountering an ADR was 26.2%, assisting in the management of ADR was 17.8% and reporting ADR was 7.5% out of the 107 respondents.

**Table 4:** Respondents’ ADR activities in the past six months.

Practices	Response	n (%)
Within the past 6 months I have encountering a patient with ADR in CCTH (N = 107)	Yes	28 (26.2)
	No	79 (73.8)
I filled and forwarded ADR form (N = 28)	Yes	8 (28.6)
	No	20 (71.4)
Within the past 6 months I have helped manage ADR patient in CCTH (N = 28)	Yes	19 (67.9)
	No	9 (32.1)

### Drugs involved in ADRs

Table 5.

**Table 5:** Drugs that have typically produced ADRs in the past 6 months.

Drugs that have typically resulted in ADRs in the past 6 months	n (%)
Antibiotics	12 (40.7)
Antihypertensives	4 (14.8)
Analgesics	7 (22.2)
Anti-diabetics	2 (7.4)
Vaccines	4 (14.8)

N = 30

### Age categories of patients who experienced ADRs and the nature of the reactions

Adults (17–63 years) constituted the highest age category that experienced adverse drug reactions. Also, most of the reported ADRs could be categorized as cutaneous reactions (62%) and most of the patients who experienced the ADRs were appropriately managed and discharged without any disability (71.4%).

### Factors that could improve ADRs reporting

According to the results as presented in Table 6, the factor that most respondents believed could improve the reporting of ADRs was continuous professional education, training, and refresher courses (95.1%) for health professionals. Also, the present study found that about 83.5% of the respondents agreed that introducing pharmacovigilance courses in the undergraduate curriculum of health professionals will help improve awareness and reporting rates of ADRs. In addition to this, continuous reminders by the national pharmacovigilance authority, promoting online ADR reporting, and increasing publications on pharmacovigilance in scientific journals are other suggestions that most respondents agreed would improve ADR reporting in Ghana. Most participants, however, disagreed with the assertion that keeping the identity of the prescribers and the reporters anonymous will improve reporting rates (Table 6).

**Table 6:** Factors that could improve reporting of ADRs.

Factor	Improves ADR reporting (%)	Does not improve ADR reporting (%)
Continuous professional education, training and refresher courses	95.1	4.9
Introduction of pharmacovigilance and ADR reporting into the pharmacy curriculum	83.5	16.5
Reminders and increase awareness from the National Pharmacovigilance Centre	88.3	11.7
Publicity about ADR reporting in local scientific journals	80.6	19.4
Designated ADR contact person in every hospital	88.3	11.7
Introduce mobile apps and on-line reporting of ADRs	84.5	15.5
Keeping the identity of reporter and prescriber a secrete	58.3	41.7

N = 107

### Associations/relationships between demographic factors and SADRR using binary logistic regression

The results obtained from the association analyses using binary logistic regression revealed that in the past 6 months, nurses were 1.22 times more likely to encounter a patient with ADRs as compared to medical doctors and they were 2 times more likely to fill and forward ADR form during management as compared to doctors (Table 7). It also revealed that in the past 6 months, health workers with more than 6 months but less than 1 year of practice experience were more likely (AOR = 1.38, 95% CI: 2.72–7.3) to meet a patient with ADRs as compared to those with the least period of practice (6 months). On the other hand, those with 6–10 years of practice experience were less (AOR = 0.86, 95% CI:0.15–1.76) likely to encounter a patient with ADRs as compared to those with 6 months of practice experience. In terms of encounter a patient with ADRs, there was no relationship between those with the least period of practice and those with the highest period of practice.

**Table 7:** Associations/relationships using binary logistic regression.

Health category	AOR (95%CI)	
**Within the past 6 months I have encountered a patient with ADR in CCTH** Doctors (ref)	1	
Nurses	1.22 (0.5–3.1)	
**Period of practice**		
6 months (ref)	1	
>6 months but <1 year	1.38 (2.72–7.3)	
1–5 years	1.18 (0.28–4.98)	
6–10 years	0.86 (0.15–4.76)	
>10 years	1	
**Sex**		
Female (ref)	1	
Male	2.42 (1.00–5.85)	
**N = 107**	
**I filled and forwarded ADR form during management**	
**Health worker category**		
Doctors (ref)	1	
Nurses	2 (0.31–2.51)	
**Gender**		
Female (ref)	1	
Male	0.49 (0.91–2.6)	

N = 28

With regards to the gender of respondents, male health workers were more likely (AOR = 2.42, 95% CI: 1–5.85) to encounter patients with ADRs as compared to the female health workers, however, they were less likely (AOR = 0.49, 95% CI: 0.91–2.6) to fill and forward ADR form during management.

Despite the observed trends in the logistic regression analysis, gender, duration of service and occupation of respondents did not significantly influence whether they would encounter an ADR or forward filled ADR forms to the appropriate authority (Table 7).

## Discussions

Adverse drug reactions (ADRs) constitute a significant cause of illness, disability and death globally. The task of detection, assessment, comprehension as well as prevention of ADRs or other drug-related mishaps are referred to as pharmacovigilance [29] and ADR reporting remains the pillar of pharmacovigilance and patient safety [17]. Conducting such studies provides very vital information that could significantly improve pharmacovigilance and medication safety in any country. There is paucity of literature on ADR reporting in Ghana and evidence from the few studies available suggests that underreporting is prevalent. To gain insight into some of the possible causes of ADR underreporting, we assessed the knowledge, attitude and practices of doctors and nurses at the Cape Coast Teaching Hospital (CCTH) on spontaneous reporting of ADR in the present study.

Having more nurses participating in this study compared to physicians is largely attributable to the fact that nurses are generally more in number than doctors at the CCTH like in many other hospitals around the world [30, 31]. Also, majority (60.7%) of the respondents were females which could be due to the fact that majority of the respondents were nurses and it is well known that the nursing profession is dominated by females unlike the medical profession [17]. The study on global gender distribution of nurses from 2000 to 2018 indicated that there are 65% of female nurses and 35% of male nurses in the African region [32] and this supports the gender distribution in this study. Another interesting finding from the demographics was that about 83% of the respondents were below 35 years of age. This agrees with an earlier study by Sabblah et al. [19] who investigated ADRs among doctors in the Greater Accra region of Ghana. It was found in that study that most of the doctors who participated in the study were young (<40 years). It is worth mentioning that the age of healthcare professionals plays an important role in their response to ADR. In fact, according to an earlier study, ADRs were three times more likely to be reported by healthcare workers who were 36–65 years of age (OR = 3.068, 95% CI 1.433–6.568) than by their younger colleagues in the 21–35 year age group [29]. With that notwithstanding, majority (71.0%) of the respondents (76 out of 107) had practiced for a minimum of one year at the time of administration of the study instruments and the remaining 29% had practiced for a minimum of 6 months (Table 1).

Data from this study also revealed that majority of the respondents had an adequate level of knowledge of ADR reporting as far as healthcare workers (82.3%) or patients (98%) report of ADRs are concerned. Their knowledge on the remaining four items was however inadequate (less than 80%). Overall, the knowledge levels of physicians and nurses at CCTH on ADR reporting could be said to be inadequate [28]. Contrasting the current findings with earlier studies, Amedome and Dadson [20] reported that 73% of healthcare professionals who took part in their study shared the perception that the main role of healthcare workers in pharmacovigilance was to report ADRs to regulatory bodies. This fell below the results obtained in our study on a similar text item. However, in another study by Ezeuko et al. [33] in Nigeria, appreciable percentage (85.8%) of healthcare workers believed ADR reporting was their professional responsibility which agreed with a similar text item in our current study. Another study by Ganesan et al. [34] in India reported that 70% of physicians and 67% of nurses saw ADR reporting as an obligation. However, on the knowledge of ADR reporting scheme in the hospital, almost half of the respondents (43.9%) did not have knowledge about its existence and where they could submit the already filled forms. It could be inferred from this results that despite the fact that the doctors and nurses who responded to the questionnaire had significant knowledge about the fact that ADR reporting is their responsibility, most of them had inadequate knowledge about where to obtain the forms and where to submit the completed forms. This is a notable reason that explains why ADRs remain under-reported at the CCTH and perhaps in other healthcare facilities in the country.

On the attitudes of the respondents, majority agreed that underreporting was due to complacency and the lack of adequate training of healthcare professionals. Majority, however, disagreed that it was due to factors such as fear of litigation, lethargy of healthcare workers and the insignificance of the ADRs reported. It is worth noting that respondents’ attitudes towards spontaneous reporting of ADRs is not different from the reasons given in other studies conducted in Ghana [18, 19]. For instance, in the study of ADRs reporting in Kpone-Katamanso District, Ghana, Asiamah and his colleagues observed that knowledge, feedback from FDA, uncertainty about cause of ADR, severity of ADR, age of respondents, fear of legal consequences, time constraint, pharmacovigilance training and unavailability of reporting forms were found to be significantly associated with spontaneous reporting of ADR [18]. Another study conducted in Uganda by Katusiime et al [29] reported similar findings, suggesting a lack of training on reporting technique as the most implicated factor accounting for underreporting of ADRs. All these studies agree with our present study and this calls for increased training of Ghanaian healthcare workers on the importance ADR reporting.

We also found that 28 out the 107 (26.2%) respondents indicated that they had encountered ADR at the CCTH within the past six months prior to the study. Out of the 28 respondents who had encountered ADR in the facility within the past 6 months, 19 (67.9%) of them were involved in the management of the ADRs whiles only 8 (28.6%) of them filled and forwarded the ADR form. This implies that the prevalence of encountering an ADR was 26.2%, assisting in the management of ADR was 17.8% and reporting ADR was 7.5% out of the 107 respondents. This prevalence is very low compared to the findings of other studies conducted in Ghana, Africa and Asia. For instance, a study by Sabblah et al. [19] in the Greater Accra Region of Ghana showed that even though more than half of the doctors interviewed had encountered a patient with an ADR over the preceding year, only 20% (less than a quarter) went ahead to complete and forward ADR reporting forms to the appropriate monitoring departments. Another study by Ganesan et al. [34] carried out in South India reported similar findings. Their study showed a rather improved prevalence where 52% of doctors, and 25% of nurses had reported the ADRs they had witnessed to an ADR monitoring center. But in this present study as presented in Fig 1, nurses at the CCTH were found to have reported more ADRs than medical doctors. However, Ganesan et al. [34] found the opposite to be true as they observed doctors to have reported more ADRs than nurses. The reasons behind our observation could be complacency, lethargy and lack of adequate training as outlined in Table 3.

In an earlier study by Mouton et al. [6], ADRs were found to contribute to the death of 2.9% of patients on admissions, of which 16% of deaths were attributable to tenofovir, rifampicin, and co-trimoxazole use. It was therefore expedient to inquire from the 28 respondents, who had encountered ADR at the CCTH within the past 6 months, to list the drugs that caused the ADRs. In all, five drug classes were identified comprising antibiotics (40.7%), analgesics (22.2%), antihypertensives (14.8%), antidiabetics (7.4%) and vaccines (14.8%) as shown in Table 5. Comparing the results with other published research, Cliff-Eribo et al. [35] studied the ADR reports submitted to the WHO Global ADR database by the Ghana National Centre for Pharmacovigilance, for children aged 0–17 years between 2000–2012. Their findings revealed that drug classes most frequently reported were vaccines (31%), antimalarials (28%) and antibiotics (15%). The variations in the types and frequencies of drug classes between our study and that of Cliff-Eribo et al. [35] study could be attributed to the disparities in ages of the populations of both studies. Cliff-Eribo et al. [35] concentrated on children below the ages of 17 and that population is known to receive a lot of vaccines, antimalarials and antibiotics unlike the predominantly adult population that was featured in our study who are noted for using analgesics, antihypertensives and antidiabetics. The findings of this study also differed from those obtained by Ampadu et al. [36] whose findings showed that ADRs from antiretroviral agents were the most common in Africa, accounting for more than 30% of African reports with nucleoside reverse transcriptase inhibitors being the greatest contributors. Moreover, ADRs due to antibiotics contributed to just 2.26% of the individual case safety results from Africa. The non-existence of ADR from antiretrovirals in the present study could be attributed to the improved regimen of antiretrovirals in recent years coupled with declining rates of HIV incidence in Ghana [37]. As presented in Table 8, majority (53.6%) of the patients who experienced ADRs fell within the 17–63 years age category. This was followed by those below the ages of 17 years (32.1%) and the elderly who were above 63 years (14.3%). This compares favorably with the results obtained by Ampadu et al. [36]. In that study, the 18–44 years age group dominated individual case safety reports from Africa, while the 45–64 years age group dominated the rest of the world. The organ system classes that were implicated in the reported ADRs were cutaneous (62.1%), gastrointestinal (31.0%) and central nervous system (3.4%). However, according to Ampadu’s [36] study, the main organ system classes reported from Africa versus the rest of the world included skin and subcutaneous tissue disorders (31.14% vs. 19.58%), general disorders and administration site conditions (20.91% vs. 30.49%), nervous system disorders (17.48% vs. 19.13%) and gastrointestinal disorders (16.10% vs. 17.86%). Interestingly, all the systems identified in our study can be found in the top 5 adverse effects reported in Africa and the rest of the world by Ampadu et al [36]. This suggests that the pattern of adverse reactions in Africa and the rest of the world has not changed significantly since 2016 and they are in sync with the data from CCTH.

**Table 8:** Age categories of patients who experienced ADRs and the nature of the reactions.

**Category of patients with ADRs**	**n (%)**
Elderly (>63 years)	4 (14.3)
Adults (17–63 years)	15 (53.6)
Children (below 17 years)	9 (32.1)
N = 28	
**Common patterns of ARDs for the past 6 months**	**n (%)**
Cutaneous	18 (62.1)
Gastrointestinal	9 (31.0)
CNS	1 (3.4)
N = 28	
**Reported ADRs in the past 6 months**	**n (%)**
The patient suffered no harm and was appropriately managed and discharged	20 (71.4)
The patient suffered severe disability from the ADR	5 (17.9)
The patient died as a result of complications from the ADR	3 (10.7)

N = 28

This study also revealed that the factor that most respondents believed could improve reporting of ADRs was continuous professional education, training, and refresher courses (95.1%), which is in concord with the study by Mouton et al, [6]. From that study, it was discovered that only 23% of respondents had ever attended a CPD or training programme on ADR reporting. Another study by Oshikoya and Awobusuyi [38] reported similar findings concerning continuous professional development and refresher courses on pharmacovigilance and ADR reporting as a crucial mechanism to improve spontaneous ADR. In another closely related study, Asiamah et al. [18] recently reported that training on pharmacovigilance significantly improves ADR reporting as healthcare professionals who received some form of training on pharmacovigilance were 5.5 times more likely to report an adverse reaction than those with no training on pharmacovigilance.

Furthermore, the results obtained from the association analyses using binary logistic regression revealed that in the past 6 months, nurses were 1.22 times more likely to encounter a patient with ADRs as compared to medical doctors and they were 2 times more likely to fill and forward ADR form during management of ADRs as compared to doctors. The results deviate sharply from the observation from an earlier study by Katusiime et al. [29] who found that compared to doctors, nurses were less likely to have reported an adverse event (OR = 0.276, 95% CI 0.117–0.650). The current findings also deviate from the study conducted by Santosh et al. [39] who also found a high percentage of doctors (82.7%) encountering ADRs compared to 67.4% by nurses during their routine work. Also, Santosh et al. [39] found that more doctors (21.6%) had ever reported ADRs to the appropriate authorities compared to nurses (17.0%). The deviation of the current study from many other studies elsewhere could be accounted for by healthcare facility specific factors that could influence ADR reporting. For instance, a Ghana-based study by Sabblah et al. [19] found that doctors working in government hospitals were about 5 times more likely to report ADRs than those in private hospitals [OR = 4.94, 95%CI (1.55–15.69)]. This suggests that even within the same profession, ownership of the healthcare facilities could have an influence on ADR reporting. It is therefore important that further studies on the subject at the CCTH should explore the reasons behind the observation.

In terms of encountering a patient with ADRs, there was no significant difference between those with the least period of practice (6 months) and those with the highest period of practice (greater than 10 years of practice). However, healthcare workers who had practiced for more than 6 months but less than 1 year period of practice were the most likely group to encounter patients with ADRs whiles those with 6–10 years practice period were the least group to encounter patients with ADRs. This disagrees with another study which found that compared to those with at most 5 years of experience, healthcare workers with more than 10 years of experience were 4 times more likely to report an adverse drug reaction [29]. Issues of complacency, lethargy to report and lack of frequent training on ADRs as found in our study might have negatively affected the ADRs reporting practices of those who had practiced for more than 10 years.

With regards to the gender of respondents, male healthcare workers were more likely (AOR = 2.42, 95% CI: 1–5.85) to encounter patients with ADRs as compared to the female healthcare workers, however, they were less likely (AOR = 0.49, 95% CI: 0.91–2.6) to fill and forward ADR form during management. Despite the fact that data on gender differences on ADR reporting is quite scanty, an earlier finding from a related study agrees with the present findings. In that study, it was observed that despite the higher proportion of serious and fatal ADRs reported by men, global post-marketing surveillance data on spontaneous reports indicate that women report more ADRs [40]. In another study, sex was found to be significantly associated with ADR reporting practice of physicians. In fact, female doctors were 3.5 times more likely to report ADR cases to national pharmacovigilance center as compared to male doctors (AOR = 3.51, 95% CI: 1.76–7.03) which was similar to our current study [41]. This suggests that men generally prefer to report serious and severe adverse drug reactions whiles women are more likely to report all forms ADRs.

This study derives its strength from the fact that it is the first of its kind in the Central Region of Ghana, thus provides useful information that could help in designing policies that could help improve spontaneous adverse drug reaction reporting in the country. Obtaining ADR reports from such under-represented populations would be vital in global pharmacovigilance Also, it details the association of demographic factors with the likelihood of encountering an ADR, managing the ADR encountered as well as filling and forwarding an ADR form to the appropriate authority.

Despite the outlined strength of the paper, it is important to recognize some important limitations of this study that need to be considered. One of such limitations is recall bias. We reduced this by exploring only ADRs which occurred within the last six months of their practice. Another limitation of our study may be related to the relatively small number of physicians and nurses who responded to the questionnaire. As such widespread generalization of study findings to the entire Central region should be done with care. Also, the small number of participants who indicated that they had encountered ADRs made the Chi-square analysis (as presented in Tables 9 and 10) statistically insignificant. This, however, does not completely rule out the observed trends in this study. A nationwide cross-sectional survey is therefore recommended.

**Table 9:** Chi-square analysis of the possibility of respondents encountering patients with ADR in the past 6 months.

Statement: Within the past 6 months I have encountered a patient with ADR in CCTH				
Parameter	Yes	No	χ2	p-value
**Gender**			1.19	0.28
Female	19	46		
Male	9	36		
**Period of practice**			2.66	0.62
6 months	3	10		
>6 months but < 1 year	6	13		
1-5yrs	15	38		
6-10yrs	4	16		
more than 10yrs	0	5		
**Occupation**			1.25	0.52
Doctor	9	31		
Nurse	19	50		

N = 28

**Table 10:** Chi-square analysis of respondents who forwarded ADR forms to authorities.

Statement: I filled and forwarded ADR form during management				
Parameter	Yes	No	χ2	p-value
**Gender**			0.15	0.7
Female	5	14		
Male	3	6		
**Period of practice**			3.83	0.28
6 months	1	1		
>6 months but < 1 year	1	5		
1–5 years	3	12		
6–10 years	3	2		
**Occupation**			0.56	0.45
Doctor	2	8		
Nurse	6	12		

N = 28

## Conclusions

Physicians and nurses who participated in the study largely exhibited inadequate levels of knowledge about existing spontaneous ADR reporting systems. Gender, period of practice, profession had an impact on whether a physician or a nurse would encounter an ADR, manage ADR or fill and forward an ADR form to the appropriate authority. Antibiotics, antihypertensives, analgesics, antidiabetics and vaccines were the common drugs implicated in ADRs within the study period. The ADRs reported mostly affected the gastro-intestinal tract, cutaneous and the central nervous systems. To address these challenge of ADR under-reporting, participants suggested that continuous sensitization and training of healthcare workers on spontaneous adverse drug reactions reporting, introduction of pharmacovigilance and ADR reporting into the pharmacy curriculum, designating contact persons in health facilities, introduction of mobile phone applications are some of the key interventions that could be implemented. Considering the dire repercussions of ADRs under-reporting, intensified efforts should be institutionalized to enable physicians and nurses identify, manage and report on them to the appropriate authority.

## Supporting information

S1 Appendix Research instrument (questionnaires). (DOCX)

S1 Data (SAV)
