# Supplementary material for: Knowledge, attitude and practice of physicians and nurses at the cape coast teaching hospital in the Central Region of Ghana on spontaneous adverse drug reaction reporting
Source: PLoS One. 2023 Jul 7;18(7):e0288100. doi: 10.1371/journal.pone.0288100 (PMC10328237; doi:10.1371/journal.pone.0288100)
Supplement: S1 Appendix — (DOCX) [file pone.0288100.s001.docx]

**APPENDIX 1**

**Research Instrument**

**UNIVERSITY OF CAPE COAST**

**COLLEGE OF HEALTH AND ALLIED SCIENCES**

**SCHOOL OF MEDICAL SCIENCE**

**QUESTIONNAIRE**

**Date: Questionnaire Number:**

**PROJECT TOPIC: Adverse drug reaction reporting by health workers of the Cape Coast Teaching Hospital.**

**A. DEMOGRAPHICS**

| **QUESTIONS** | **OPTIONS** |
| --- | --- |
| 1. Age | - 18 – 25 - 26 - 35 - 36 – 45 - 46 – 55 - 56 – 65 - > 65 |
| 2. Gender | - Female - Male |
| 3. Department | - Medicine - Surgery - Obstetrics and gynecology - Pediatrics - Family medicine - Accident and emergency - Ophthalmology - ENT - Orthopedics - Anesthesia - Dentistry - Others (specify)……………………… |
| 4. Occupation | - Doctor…………………if yes please move to (5) - Nurse………………… if yes please move to (6) - Pharmacist……………if yes please move to (7) - Physician assistant…...if yes please move to (7) |
| 5. Category of Doctor | - Consultant - Senior Specialist - Specialist - Resident - Medical Officer - House Officer |
| 6. Category of Nurse | - Community Health Nurse - Enrolled Nurse - Midwife - Professional Nurse |
| 7. Period of practicing in Cape Coast Teaching Hospital | - 6 months - 7 months – 9 months - 1 year – 5 years - 6 years – 10 years - >10 years |

**B. KNOWLEDGE OF HEALTHCARE WORKERS ON ADR REPORTING**

| Questions | Strongly Disagree | Disagree | I don’t know | Agree | Strongly Agree |
| --- | --- | --- | --- | --- | --- |
| 8. Healthcare workers are obliged by law Public Health Act 2012, Act 851, Part 7, Section 125, to report Adverse drug reactions (ADRs) |  |  |  |  |  |
| 9. Individuals can also report Adverse drug reactions |  |  |  |  |  |
| 10. The yellow card scheme is currently used in Nigeria and the UK to promote ADR reporting |  |  |  |  |  |
| 11. In Ghana, patient reporting forms are available for reporting ADRs |  |  |  |  |  |
| 12. Adverse drug reactions (ADR) resulting from non-prescription and prescription drugs (including biological products and radiopharmaceutical products)must be reported |  |  |  |  |  |
| 13. Adverse reactions resulting from herbal medicinal products and food supplements medical devices, cosmetics and household chemical substances should not be reported |  |  |  |  |  |
| 14. Adverse reactions occurring in a recipient of blood or blood components do not constitute ADRs |  |  |  |  |  |
| 15. Drug abuse, drug overdose, drug interactions, quality defects, poor packaging, questionable stability, suspected contamination, suspected counterfeit and lack of therapeutic efficacy do not constitute ADRs |  |  |  |  |  |
| 16. All serious suspected and serious unexpected adverse drug reactions associated with the use a product in Ghana should be reported to the Authority within 7 calendar days. |  |  |  |  |  |
| 17. All other adverse drug reactions will be reported to the Authority within a period of 28 days.  18. The National Pharmacovigilance Centre is located in Kumasi |  |  |  |  |  |
| 19. I am aware of where I might access forms in my institution in other to report an ADR |  |  |  |  |  |
| 20. I know where to return the fully filled ADR reporting forms |  |  |  |  |  |

**C. PRACTICE OF ADR REPORTING**

| **Questions** | **Agree**  **(1)** | **Disagree**  **(2)** |
| --- | --- | --- |
| 21. Within the past 6 months I have seen a patient with an ADR at CCTH  If yes please move to question 22, if no jump to the next section on attitudes |  |  |
| 22. Within the past 6 months I have been part of the management (in –patient/out- patient)of a patient with an ADR in CCTH……..  If you agree please move to question (23 and 24) |  |  |
| 23. As part of the management of this patient, I filled an ADR reporting form and forwarded it in accordance with the established protocol |  |  |
| 24. As part of the management of this patient, another member of the team filled the ADR form and forwarded the form in accordance to the established protocol |  |  |
| 25. The patient suffered severe disability from the ADR…………………  If you agree (1) please specify the disability ………………………… |  |  |
|  |  |  |
| 26. The patient died as a result of complications of the ADR………………  If you agree (1) please specify the complication………………………. |  |  |
| 27. The patient suffered no harm, was appropriately managed and discharged |  |  |

| **Questions** | **Options (please tick the options applicable)** |
| --- | --- |
| 28. In the past 6 months of your practice, what categories of patients have been most affected by ADRs? | - Adults (>16 years) specify age……………. - Children (from birth to 16years)………….. - Males - Females |
| 29. In the past 6 months of your practice which of the following patterns of ADRs have been encountered by you? | **A. Cutaneous manifestations**   - Steven Johnson’s Syndrome - Erythema multiformes minor - Acute Generalized Exanthematous pustulosis - Fixed drug eruption - Drug induced Pemphigus - Drug induced Pemphigoid - Toxic Epidermal Necrolysis - Other (specify)………………………   **B. Gastrointestinal manifestations**   - Acute Diarrhoea - Acute Constipation - Abdominal Pain - Dyspepsia - Anorexia - Other (specify)………………………   **C. Central Nervous System**   - Head aches - Tremors - Paresthesia - Tardive dyskinesia - Fever - Other (specify)………………………   **D. Endocrine**   - Hypoglycaemia - Other (specify)……………………… |
| 31. In the past 6 months which drugs have typically resulted in ADRs( after selecting the drug class, state the specific drug) | - Antibiotics ………………………………….. - Antihypertensive drugs……………………… - Analgesics……………………………………. - Anti-diabetic drugs…………………………… - Herbal preparations and Supplements………… - Others…………………………………………. |

**D. ATTITUDES OF HEALTHCARE WORKERS TOWARDS ADR REPORTING**

| **Questions** | **Agree**  **(1)** | **Disagree**  (**2)** |
| --- | --- | --- |
| 32. I have attended a CPD or training programme on ADR reporting before |  |  |
| 33. Underreporting of ADRs by health workers is attributable to complacency on the part of healthcare workers |  |  |
| 34. Underreporting of ADRs by health workers is attributable to lethargy on the part of healthcare workers |  |  |
| 35. Underreporting of ADRs by health workers is to prevent legal issues and avoid financial burden |  |  |
| 36. Underreporting of ADRs by heath workers is attributable to lack of training on the technique of reporting |  |  |
| 37. Underreporting of ADRs by heath workers is attributable to fact that ADR are secondary issues and are not that significant in practice |  |  |

**E. REMARKS**

Suggestions to improve spontaneous reporting of ADR in CCTH and Ghana

……………………………………………………………………………………………........................................................................................................................................................................................................................................................................................................................................

.………………………………………………………………………………………………….…
